# Diurnal dynamics of oxygen and carbon dioxide concentrations in shoots and rhizomes of a perennial in a constructed wetland indicate down-regulation of below ground oxygen consumption

**DOI:** 10.1093/aobpla/plw025

**Published:** 2016-07-11

**Authors:** Anna C. Faußer, Jiří Dušek, Hana Čížková, Marian Kazda

**Affiliations:** 1 Ulm University, Institute of Systematic Botany and Ecology, Ulm, Germany; 2 CzechGlobe - Global Change Research Centre AS CR, Department of Matters and Energy Fluxes, v.v.i. České Budějovice, Czech Republic; 3 University of South Bohemia, Faculty of Agriculture, Department of Biology, České Budějovice, Czech Republic

**Keywords:** Aeration, constructed wetland, in-situ field study, internal carbon dioxide, internal oxygen dynamics, *Phragmites australis*

## Abstract

Wetland plants actively provide oxygen for aerobic processes in submerged tissues and the rhizosphere. The novel concomitant assessment of diurnal dynamics of oxygen and carbon dioxide concentrations under field conditions tests the whole-system interactions in plant-internal gas exchange and regulation. Oxygen concentrations ([O_2_]) were monitored *in-situ* in central culm and rhizome pith cavities of common reed (*Phragmites australis*) using optical oxygen sensors. The corresponding carbon dioxide concentrations ([CO_2_]) were assessed via gas samples from the culms. Highly dynamic diurnal courses of [O_2_] were recorded, which started at 6.5–13 % in the morning, increased rapidly up to 22 % during midday and declined exponentially during the night. Internal [CO_2_] were high in the morning (1.55–17.5 %) and decreased (0.04–0.94 %) during the rapid increase of [O_2_] in the culms. The observed negative correlations between [O_2_] and [CO_2_] particularly describe the below ground relationship between plant-mediated oxygen supply and oxygen use by respiration and biogeochemical processes in the rhizosphere. Furthermore, the nocturnal declining slopes of [O_2_] in culms and rhizomes indicated a down-regulation of the demand for oxygen in the complete below ground plant-associated system. These findings emphasize the need for measurements of plant-internal gas exchange processes under field conditions because it considers the complex interactions in the oxic-anoxic interface.

## Abstract (precis)

Plants have evolved mechanisms to provide oxygen to their parts in oxygen-free environments like wetland sediments. We measured the diurnal courses of oxygen supply to rhizomes of the common reed, a widespread wetland plant. During the day the below-ground plant parts can rely on ample oxygen, but during the night its supply to rhizomes and roots as well as to the whole assembly of associated microorganisms is limited. The key finding of the study was that during periods of low oxygen supply the whole below-ground biota reduces its respiration. This regulation mechanism helps the biota survive unfavourable periods.

## Introduction

Under strongly anoxic conditions in permanently water-saturated soils of wetland ecosystems (Ponnamperuma 1972), oxygen is a very rare resource with limited occurrence and, if present, it is immediately utilized by biological and chemical processes. Plants growing under such conditions frequently develop an enlarged gas-space continuum which runs from shoots through the extensive rhizome system to the roots (Cardoso *et al.* 2014; Jackson and Armstrong 1999; Končalová 1990). This aerenchyma system enables emergent wetland plants to supply submerged organs with oxygen. In return respiratory gases (e.g. CO_2_) from below ground aerobic, anaerobic and fermentation processes (e.g. aerobic respiration, denitrification, iron reduction) as well as methane from the anaerobic sediments can ventilate to the atmosphere (Armstrong 1980; Armstrong *et al.* 2000; Kayranli *et al.* 2010; Le Mer and Roger 2001; Laanbroek 2009; Maltais-Landry *et al.* 2009).

Wetland plants enhance their internal ventilation capacity by pressurized gas-flow through the aerenchymatic tissue (Armstrong and Armstrong 1991; Brix *et al.* 1992). Pressurization is achieved by the generation of gradients of temperature (thermal-transpiration) and/or humidity (humidity-induced convection) over a porous partition. The following free flow of gas inside the aerenchymatic tissue is realized under Knudsen regime between the inner and outer atmosphere (Armstrong *et al.* 1996). Through stems and rhizomes, gas-flow in *Phragmites australis* can also occur as a result of wind blowing over the stand by which air pressure gradients (Venturi effects) are created between young and old or dead culms (Armstrong *et al.* 1992). In *P. australis* gas through-flow occurs mainly via the pith cavity, as the cortex cylinder has very low porosity (Afreen *et al.* 2007).

The described ventilation mechanisms can occur simultaneously, but prevailing pressurization types vary between species. Pressurization in emergent wetland macrophytes with graminoid growth type is mainly driven by humidity-induced convection (e.g. *Eleocharis spacelata*, *Typha sp*., *Juncus ingens*, *P. australis*). In parallel, pressurization efficiency is affected by other factors including light intensity, shoot height, leaf sheath area, photosynthesis and stomatal aperture (Afreen *et al.* 2007; Armstrong and Armstrong 1991; Bendix *et al.* 1994; Brix *et al.* 1992, 1996; Tornbjerg *et al.* 1994). The combined effects of pressurization processes can result in internal overpressure between 4 and 11 hPa (Afreen *et al.* 2007; Armstrong and Armstrong 1990), which sustains the internal oxygen concentration ([O_2_]) in submerged organs close to ambient levels (Brix *et al.* 1996; Konnerup *et al.* 2011).

Most roots of wetland plants develop barriers to prevent radial loss of oxygen to the soil (ROL) by impeding radial diffusion in the epidermal and hypodermal cell layers (Armstrong *et al.* 2000; Soukup *et al.* 2002). Nonetheless, oxygen is released into the rhizosphere through porous regions at the root tips, lateral roots and through gas permeable ‘windows’ opposite of developing laterals (Armstrong *et al.* 2000). Thus, partly oxic conditions occur in the rhizosphere (Mainiero and Kazda 2005), which allow oxidation of the phytotoxic compounds, which characterize anaerobic soils. Although aerobic soil microbes benefit from locally oxic conditions in the rhizosphere (Faußer *et al.* 2012), plants can utilize products of the mineralization processes by the rhizobacterial communities (Radruppa *et al.* 2008). In such interactions, oxygen supplied below ground is readily consumed and soil borne respiratory gases may enter the internal gas-spaces (Colmer 2003). Consequently, diurnal courses of oxygen and carbon dioxide can be observed in internal plant cavities in both above and below ground plant parts (Brix *et al.* 1996; Konnerup *et al.* 2011). It was reported that the internal partial pressure of CO_2_ in *Typha latifolia* leaves is up to 10 times elevated compared with ambient air (Constable *et al.* 1992). Laboratory experiments on rice plants showed CO_2_ concentrations ([CO_2_]) of above 9 % when growing in CO_2_ enriched media (Higuchi 1982).

During periods of limited air ventilation to the submerged organs, e.g. during the night when pressurization mechanisms are unfavourable, the level of internal [O_2_] may fall in the range of hypoxic conditions (Armstrong *et al.* 2009; Gupta *et al.* 2009). It was reported that segments of plant roots are able to down-regulate their cellular rate of respiration if the internal [O_2_] drop to <3.1 % (i.e. 45 hPa) (Armstrong *et al.* 2000, 2009; Berry and Norris 1949; Čížková and Bauer 1998; Gupta *et al.* 2009; Zabalza *et al.* 2009). Armstrong and Beckett (2011b) proposed a model for the mechanism of oxygen conservation in respiring root segments when internal oxygen levels are limited under the hypoxic conditions. In a similar way, soil microbes in the rhizosphere will regulate their cellular rate of aerobic respiration during times of limited oxygen supply (Jin and Bethke 2003).

In this study, diurnal dynamics of [O_2_] inside rhizomes and culms were connected to plant-internal [CO_2_]. Please note that a pre-version of this article is included in the doctoral thesis of the first author. At the time of writing, the thesis is not yet published and not citable. Such measurements under field conditions can reveal the whole-system interactions in plant-internal gas transport, gas exchange and oxygen conservation by regulation of its consumption. Based on this, the following three hypotheses were tested:

- Plant-internal concentrations of O_2_ and CO_2_ are negatively correlated. Despite the aeration in wetland plants is well documented (Afreen *et al.* 2007; Bendix *et al.* 1994; Brix *et al.* 1996; Colmer 2003; Konnerup *et al.* 2011; Sorrell and Brix 2003) there are only few data on plant-internal [CO_2_] (c.f. Constable *et al.* 1992). Thus the aim of this study was to provide a complementary view on the dynamics of both gases under field conditions.
- In the morning hours, [O_2_] increase rapidly and internal [CO_2_] should decline concomitantly due to photosynthesis and aeration mechanisms of the culms. We expect that these courses of [O_2_] and [CO_2_] are regular and repeatable throughout several days and can reveal patterns of the plant internal aeration capacity and regulation mechanisms.
- Below ground oxygen consumption from the internal gas spaces is the sum of plant respiration, oxygen loss to the rhizosphere (i.e. ROL) and all oxygen-dependent processes in the rhizosphere (biological and chemical). Thus a regulation of the oxygen consumption from the plant-internal gas-spaces is obvious during periods of limited oxygen supply, i.e. during night at ceasing pressurization, and can be tested experimentally by culm removal (no pressurization). Under such conditions, [O_2_] should not decline below a minimum level within a reasonable time frame. The expected decline was already proposed for root segments under laboratory conditions (Armstrong and Beckett 2011b) and shall here be tested *in* *situ* for the whole below ground plant system. The results allow us to interpret the degree of oxygen consumption and conservation by the whole submerged plant associated system and thus to contribute to the recent ‘respiratory down-regulation debate‘ (Armstrong and Beckett 2011a).

The results of the presented field study are of added value to previous experiments (Afreen *et al.* 2007; Armstrong and Armstrong 1991; Armstrong and Armstrong 2005), as they were gathered under field conditions and not in the laboratory, in pots or on excised plants. Perennials in an established constructed wetland (CW) were chosen for the study, as the environmental parameters (e.g. water level, flow rate, water quality) of this system could be well monitored. Furthermore, the growing conditions for the plants are close to those in natural wetlands, while the homogeneous substrate made of gravel is the only noteworthy difference (Dušek *et al.*, 2008; Picek *et al.* 2007). Studies under undisturbed growing conditions in a wetland ecosystem provide knowledge on interactions between plants and microorganisms (Bodelier 2003) in the rhizosphere competing for resources like nutrients and oxygen. In order to understand the whole ecosystem functioning of wetlands, it is crucial to analyze system interactions of the oxygen cycle under undisturbed conditions.

## Methods

### Site description

This study was conducted in a subsurface horizontal flow CW located in Slavošovice in South Bohemia, Czech Republic in August 2009. This CW was chosen for our study as its development and growing conditions are well documented by previous studies (Dušek *et al.*, 2008; Picek *et al.* 2007) which guaranties better interpretation of the findings. Situated at 480 m above sea level, the annual average air temperature was 7.9 °C and the annual precipitation was 634 mm. Table 1 gives meteorological data at the study site for month August. The facility consisted of a pretreatment (screen, sand trap, sedimentation tank) and two vegetated treatment beds each of 17 m length and 22 m width (total treatment area 374 m^2^) planted with common reed (*P.* *australis*). The CW was created for municipal waste water treatment of 150 person equivalents and started operation in August 2001. The vegetated part of the treatment beds was filled with fine gravel (3–20 mm) while the margins at inlet and outlet (1.5 m each) consisted of coarse gravel (50–100 mm). Water and substrate properties are characterized in detail in earlier studies (Dušek *et al.* 2008; Picek *et al.* 2007). Analysis of the final effluent showed a high efficiency of the system in removing organic pollution (reduction of biological oxygen demand, BOD_5_, by 82 % and chemical oxygen demand, COD, by 74 %), total nitrogen (63 %), total phosphorus (75 %) and suspended solids (52 %) (Dušek *et al.* 2008). On average, the waste water inflow rate was regulated to 0.12 ± 0.10 Ls^−^^1^ (mean ± SD), with a maximum of 1.0 Ls^−^^1^ during periods of extremely strong precipitation. During the vegetation season, the water level was controlled to 20–30 mm under the gravel surface.

**Table 1.** Meteorological conditions of Slavošovice CW.

Average values		Period of measurements	7 year averages
		August 2009	August 2002–09
Air temperature [°C]		14.6 ± 5.2 (mean ± SD)	14.9 ± 4.0
Water temperature [°C]	0.2 m depth	14.2 ± 1.8	14.9 ± 1.8
	0.5 m depth	14.1 ± 1.9	15.2 ± 1.9
Inflow rate [Ls^−1^]		0.18	0.30
Precipitation [mm]		69.2	45.3

Until this study in August 2009, the common reed successfully established a dense stand covering all available CW area. The stand was estimated as healthy according to the shoot density, average shoot height and appearance (cf. Dickopp *et al.* 2011). Above and below ground biomass of the common reed stand was evaluated earlier (Picek *et al.* 2007).

**Figure 1.**
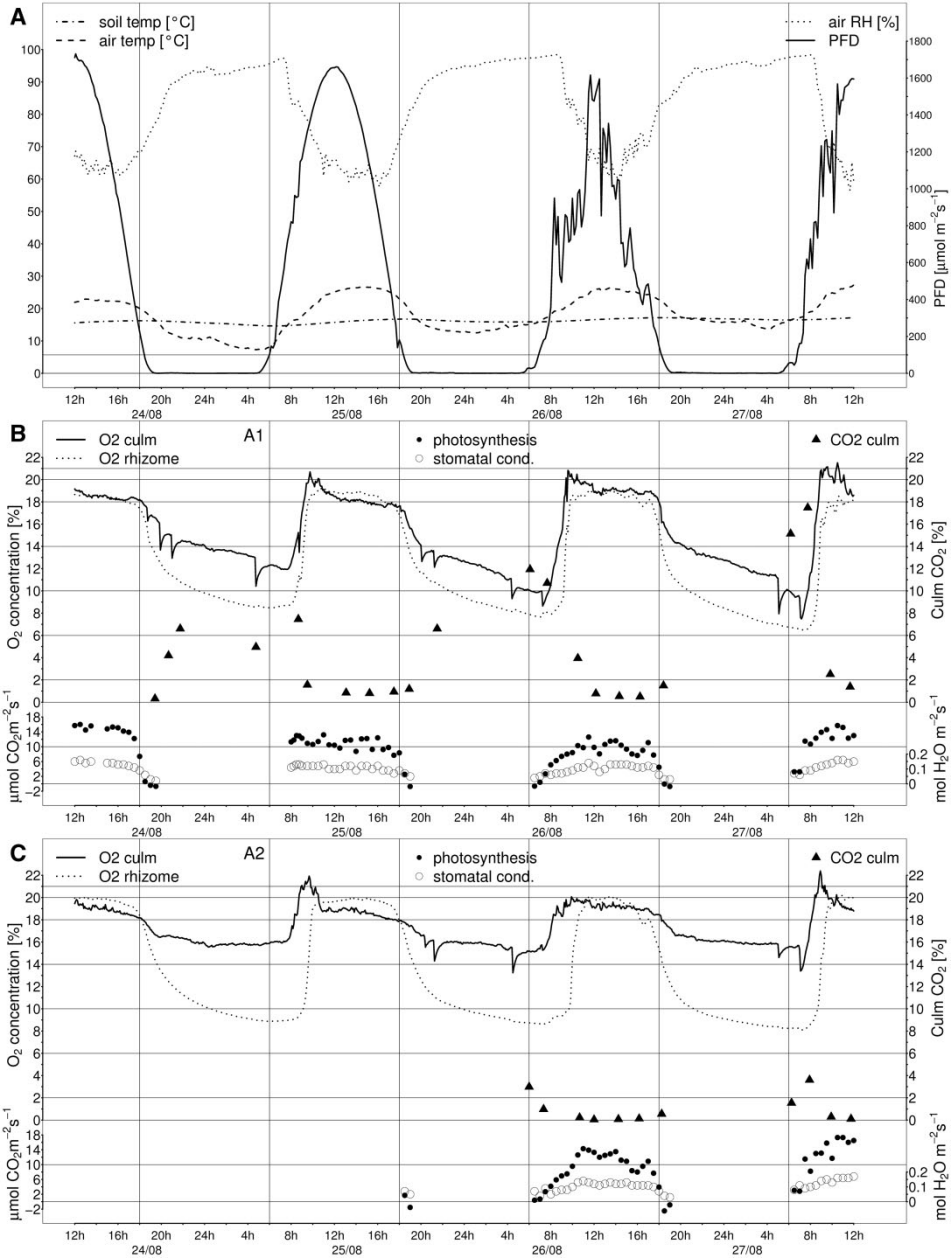
Diurnal oxygen courses in central pith cavities of culms and rhizomes of two *P. australis* plants and micro-climatic conditions during measurements **(A)**. Plants A1 **(B)** and A2 **(C)** analyzed grew in a CW in Slavošovice, Czech Republic. Optodes for recording oxygen concentration were implanted about 30 cm above the substrate in shoots and in vertical rhizome ends elevating the water table. Values were recorded continuously every five minutes over 72 h in August 2009. Time is given in CET without summer time adjustment. RH, relative air humidity; PFD, photosynthetic photon flux density.

During the period of measurements we recorded microclimatic factors inside the stand in a height of 1 m above the gravel surface. These parameters included photon flux density (PFD) over the waveband 400–700 nm, air temperature and relative air humidity (RH). PFD sensors were produced by LI-COR Biosciences, Lincoln NE, USA; temperature and RH sensors by Delta-T, Burwell, UK. Additionally, substrate water temperature of the reed bed was logged. PFD outside the stand was gathered in a height of 1.5 m. Stable weather conditions prevailed during diurnal oxygen recording (cf. Fig. 1A). In all data, time of day is presented as Central European Time (CET) without summer time adjustment.

### Plant selection

Measurements in the internal gas-spaces of reed plants were performed in two regions of the southern vegetation bed of the CW: the first half closer to the inflow and the second half closer to the outflow. Two healthy shoots of *P. australis* plants were selected for discontinuous measurements of internal oxygen concentration ([O_2_]) and carbon dioxide concentration each in the inflow region (In1, In2) and outflow region (Out1, Out2), respectively.

For intensive assessment of [O_2_] in culms and directly adjacent rhizomes, two additional plants (A1, A2) were assigned in the second half of the CW vegetation bed. In this region high amplitudes in internal oxygen were detected in rhizomes (Dickopp *et al.* 2011). The rhizome systems of the reed plants were gently excavated from leaf litter and gravel (each layer of about 50 mm in thickness). Two dead ending vertical rhizomes, with short stumps of shoots from earlier years (1–2-years old), were chosen in order to connect to directly adjacent this year’s culms. On those two plants also gas exchange measurements of top leaf blades and analysis of internal [CO_2_] were performed.

### Oxygen measurement

For the measurement of [O_2_] in the reed culms (A1, A2, In1, In2, Out1, Out2), optical oxygen sensors (diameter 4 mm, type PSt3, PreSens GmbH, Regensburg, Germany; Klimant *et al.*, 1995) were implanted into internodes (300 mm above ground) through drilled openings. On the plants A1 and A2, the ends of the corresponding rhizomes were cut open 25–55 mm above the water table (i.e. within the gravel layer) and equipped with the same type of oxygen sensor as in the culms. The generated openings in shoots and rhizomes were sealed with several layers of laboratory film (Parafilm, American National Can, Chicago, USA) subsequently after sensor implantation. Oxygen concentrations in In1, In2, Out1 and Out2 were recorded by FIBOX 3 (PreSens GmbH, Regensburg, Germany) every hour on a daily course. The use of a four channel gauge (Oxy-4 mini, PreSens, Regensburg, Germany) connected to the culms and rhizomes of A1 and A2, enabled us to monitor changes in [O_2_] continuously every five minutes for three complete diurnal courses (24–27 August 2009). During measurements the excavated rhizome systems were covered with aluminium foil to protect them from any direct sunlight, which might penetrate the reed stand. Data recorded from the sensors were recalculated to values of [O_2_] according to the temperatures of air (for values in culms) or in water (for values in rhizomes), which were assessed close to the implanted optodes. The readings of [O_2_] were combined with the below described [CO_2_] observations to test their negative relationship stated in hypothesis 1.

### Carbon dioxide measurement

Concentrations of CO_2_ were measured with a mobile gas analyzer based on laser absorption technology (Los Gatos Research, Inc., USA, Model: 908-0007). The air samples (10 mL) from inside the reed culms were taken by a gas-tight syringe from the internodes above the oxygen sensor insertion position. The syringe needles were mechanically fixed and sealed with laboratory film to reduce possible leakage of air out of the reed culms. Any manipulation was limited to the sampling procedure only. The needles were closed by empty syringes or by rubber caps during each interval between two sampling times. Immediately after sampling, the air samples were analyzed on the laser gas analyzer. The [CO_2_] measurements were performed on average every 2 h during daylight.

### Photosynthetic gas exchange measurement

In order to observe the activity of the investigated reed plants A1 and A2, the rate of photosynthetic assimilation (A) and stomatal conductance (g_s_) were assessed. Correlations between the plant-internal [O_2_] and the rate of photosynthesis and microclimatic parameters were computed to test the influence of plant activity on ventilation mechanisms (hypothesis 2). Gas exchange measurements were performed every half an hour on leaf blades (second from the top inflorescence) using a LI-6400 Portable Photosynthesis System (LI-COR Biosciences, Lincoln NE, USA) at ambient conditions of light and CO_2_. It was observed that the leaf blades turned towards the sun during the day. Thus the measurement chamber was arranged carefully with the turning leaf blades in direction of the sunlight to gather maximum PFD on the leaves.

### Experimental limitation of oxygen supply

At midday (12:15 h) on the final day of [O_2_] measurement, culms A1 and A2 were cut at the internodes above the oxygen sensor insertion position and sealed with laboratory film. Oxygen measurements in culms and rhizomes continued for the next 20 hours, in order to monitor the plant-internal [O_2_] when no more pressurization is possible. This allows to observe if there is any conservation of plant-internal oxygen as the result of regulation mechanisms in the whole submerged system during limited oxygen supply (hypothesis 3).

### Statistical analysis

Relationships between [O_2_] and gas exchange measurements (Table 2), between [O_2_] and microclimatic parameters (PFD, temperature of air and surface water, RH; Table 2), and between [O_2_ and CO_2_] (Table 4) were evaluated to statistically test the hypotheses 1 and 2. The correlation coefficients were calculated based on the Pearson’s product moment correlation (*r*) with subsequent test for significance (R function ‘cor.test’, R Development Core Team 2010). For the significance tests, the degree of freedom is df =  n – 2 and the *t*-value is $t=r\;/\sqrt{\frac{1-r^{2}}{n-2}},\;$where ‘*n*’ is the number of instances and ‘*r*’ is the correlation coefficient. The *P*-values were retrieved from a t-distribution, using the df and *t*-values. In the result tables, the correlation coefficients *r*, the degrees of freedom df and the *P*-values are reported. The correlations were calculated for each plant individually and for the whole data set consisting of six plants. We considered a correlation to be significant when the *P*-value was below 0.05.

**Table 2.** Correlations (Pearson’s product moment) between O_2_conc in culms and rhizomes of A1 and A2 and microclimatic factors and gas exchange. The worst of the two calculated correlations of the investigated plants is given. Calculations for RH (relative air humidity), air temperature (Air T) and water temperature (Water T) included measurements 12:00–24:00 h on 24 August 2009 (df = 286), 00:00–24:00 h on 25 and 26 August 2009 (df = 286 each), and 00:00–12:00 h on 27 August 2009 (df = 143). Correlations with PFD (photon flux density) were evaluated during sun hours: 05:00–19:00 h (i.e. 12:00–19:00 h on 24 August 2009 (df = 167) and 05:00–12:00 h on 27 August 2009 (df = 83)). Correlations between [O_2_] and photosynthesis (a) and stomatal conductance (g_S_) were calculated with the corresponding measurements (24 and 25 August 2009 df = 12; 26 August 2009; df = 24; 27 August 2009 df = 10). According to the significance tests, the correlations had *P*-values of *P* < 0.05 with the exceptions indicated as not significant (ns).

	RH	Air T	Water T	PFD	A	g_S_
Culms (*n* = 2)
24 August 2009	−0.82	0.83	0.66	0.56	0.88	0.94
25 August 2009	−0.79	0.78	ns	0.67	ns	ns
26 August 2009	−0.83	0.90	0.47	0.65	0.77	0.70
27 August 2009	−0.74	0.76	0.58	0.81	0.68	0.67
Rhizomes (*n* = 2)
24 August 2009	−0.94	0.93	0.61	0.56	0.93	0.96
25 August 2009	−0.96	0.94	0.50	0.54	ns	ns
26 August 2009	−0.95	0.93	0.52	0.65	0.78	0.79
27 August 2009	−0.97	0.94	0.81	0.92	0.73	0.92

**Table 3.** Details of morning increase of the oxygen concentrations (O_2_conc) in culm and rhizome pith cavities of *P. australis* plants A1 and A2. The times given indicate the period between the first sharp increase in O_2_conc (‘start time’) until highest values were reached and slopes flattened (‘end time’). Values of ΔO_2_conc show the extent of increase in O_2_conc. Time in [hh:mm] CET; O_2_conc in [%]; PFD, photon flux density in the open in [µmol m^−2^ s^−1^]; RH, relative air humidity inside stand in [%].

Date			Start time	PFD	RH	O_2_conc	End time	PFD	RH	O_2_conc	A O_2_conc
24 August	A1	culm	08:10	935	81	7.9	10:45	1611	70	19.6	12
		rhizome	08:20	993	80	6.8	10:10	1503	70	18.3	12
25 August	A1	culm	07:40	716	93	11.9	09:45	1368	75	20.7	9
		rhizome	07:45	739	90	8.7	09:45	1368	75	18.4	10
	A2	culm	07:40	716	93	16.2	09:40	1347	76	21.9	6
		rhizome	08:35	956	84	9.2	09:55	1409	73	18.5	9
26 August	A1	culm	07:40	231	98	9.7	09:35	778	85	20.8	11
		rhizome	09:05	585	94	9.2	09:55	837	83	18.4	9
	A2	culm	07:40	231	98	15.9	09:55	837	83	20.1	4
		rhizome	09:35	779	84	9.8	1125	1364	68	19.9	10
27 August	A1	culm	07:25	376	98	8.3	09:35	1203	70	21.2	13
		rhizome	08:10	569	98	7.0	08:55	1126	86	15.5	9
	A2	culm	07:25	376	98	14.3	08:55	1126	86	22.4	8
		rhizome	08:40	733	97	10.0	09:35	1203	71	19.0	9

**Table 4.** Pearson correlation coefficients between carbon dioxide and oxygen concentrations in the central pith cavities of six culms of *P. australis* growing in the studied CW. Two plants (In1, In2) grew in the first half of the CW and four plants (A1, A2, Out1, Out2) were located in the second half of the CW. The *P*-values, according to the subsequent tests for significance, are indicated as follows: ns not significant; *P < 0.05; **P < 0.01; ***P < 0.001. *r*, correlation coefficient; df, degree of freedom (number of observations −2); NA, not available; empty entry: not determined.

Plants	In1		In2		A1		A2		Out1		Out2	
	*r*	df	*r*	df	*r*	df	*r*	df	*r*	df	*r*	df
24 August 2009	−0.96***	6	−0.82*	6	NA		NA		−0.24 ns	6	−0.77*	6
25 August 2009	−0.62 ns	4	−0.94**	4	−0.71*	6	NA		0.61 ns	4	−0.99***	4
26 August 2009	−0.96***	5	−0.7*	5	−0.91**	5	−0.83*	5	−0.82*	5	−0.83*	5
27 August 2009	0.77 ns	4	−0.92*	4	−0.98*	2	−0.63 ns	2	−0.82*	4	−0.87*	4
whole period	−0.47*	25	−0.82***	25	−0.84***	20	−0.7*	9	−0.23 ns	25	−0.85***	25

Mean values were calculated for minimum and maximum values of [O_2_] inside the six investigated culms during the measurements. The results are presented with SD and number of included culms (*n*). A mean value, together with SD and the number of instances (*n*), is given also for the extent of the sudden oxygen drops recorded during oxygen decline during the night (see below).

### Evaluation and modelling of diurnal oxygen courses

In order to evaluate the degree of oxygen conservation mechanisms of the below ground system, the temporal courses of [O_2_] at nocturnal declines and after cutting of the culms were fitted using the exponential equation:

(1) $$f\left(t\right)=\;a\cdot \exp\left(b\cdot t\right)+\;c$$

where t is time in seconds and a, b, c∈R are the parameters to be fitted. Curve fitting was performed by minimizing the least squares with Genetic Algorithms (R, package gafit, www.R-project.org). Intercept ‘c’ in this formula indicates the minimum levels to which internal [O_2_] possibly converged when oxygen consumption is regulated. Coefficients of determination (*r*^2^) were calculated to give the degree by which the exponential curves represent the declining [O_2_] in the reed pith cavities. The gradients of the plots given by Equation (1) are expressed by its derivative:

(2) $$\frac{\partial f\left(t\right)}{\partial t}=a\cdot b\cdot exp(b\cdot t)$$

Slopes of [O_2_] are assumed to reflect the extent of oxygen consumption due to respiration in plant tissue and rhizosphere-related oxygen demand or loss when main convective ventilation mechanisms are missing overnight or after culm cut-off.

## Results

### Diurnal oxygen dynamics

The internal oxygen concentration, measured *in-situ* in culms and rhizomes, showed distinct diurnal patterns (plants A1 and A2 in Fig. 1). On a daily basis, culm-[O_2_] was correlated strongly with RH (*r* ≤ −0.74; all *p* < 0.001, see Table 2) and air temperature (*r* ≥ 0.76), and moderately with PFD (*r* ≥ 0.56). Courses of rhizome-[O_2_] in the reed plants were similar to those observed in culms and were correlated strongly to RH (*r* < −0.94) and air temperature (*r* ≥ 0.93), and moderate with surface water temperature (*r* ≥ 0.50) and PFD (*r* ≥ 0.54).

In the morning hours, minimum [O_2_] ranged in culms from 9.4 to 16.1 % and in rhizomes from 6.7 to 9.0 %. Following sunrise, between 07:25 and 07:40 h, culm-[O_2_] increased steeply (Fig. 1, Table 3). This pattern was recurrent on each morning at PFD between 200 and 900 µmol m^−^^2^s^−^^1^. At the same time, RH was mainly high but started to decline and the air temperature had risen above 15 °C. Rhizome-[O_2_] followed this steep slope subsequently or with a delay of up to 100 min. Especially on 26 August 2009, which was a more windy day with some clouds, the delay in rhizome-[O_2_] increase was more prolonged compared with the culm-[O_2_] increase.

Maximum [O_2_] values above 19.9 % were reached in the culms typically starting from 08:50 and at RH below 85 %. From 10:30 h on during high PFD and minimum RH (around 60 %), culm-[O_2_] evened out at values around 18.8 %. Maximum [O_2_] in rhizomes were reached 45–120 min later than in culms. Rhizome-[O_2_] maintained at high levels between 18.8 and 19.9 % for up to 6 h, thus oxygen concentrations were elevated in the rhizomes compared with the culms for some periods (Fig. 1).

In the late afternoon, first rhizome-[O_2_] (17:00 h) and then culm-[O_2_] (18:00 h) began to decline rapidly. Meanwhile PFD dropped below 200 µmol m^−^^2^s^−^^1^ and RH increased while air temperature was still above 20 °C. The [O_2_] decline was more pronounced in the rhizomes than in the culms, and more distinct in culm A1 compared with culm A2 (Fig. 1). During the night-time [O_2_] levelled to minimum values shortly before sunrise.

Internal [O_2_] in the culms of the other four investigated plants (In1, In2, Out1, Out2) followed similar diurnal courses than in plants A1 and A2, but as the [O_2_] assessment was only once per hour, the picture of their diurnal pattern was fragmentary compared with plants A1 and A2. Minimum morning values occurred between 07:00 and 10:00 h and ranged from 9.6 to 17.3 % in the inflow zone and from 6.1 to 19.6 % in the outflow zone. Maximum [O_2_] were recorded earlier in the inflow zone (09:00–12:00 h; 16.9 % ± 4.8 SD to 19.9 % ± 1.9) than in the outflow zone (11:00–13:00 h; 18.6 ± 2.1 to 20.1 ± 2.1).

During the late evening and early morning, several short-term [O_2_]-drops were recorded in culms A1 and A2 (Fig. 1). The change in internal culm-[O_2_] during the [O_2_]-drops ranged from −0.5 to −3.2 % (mean −1.8 ± 0.6 % SD, *n* = 17 [O_2_]-drops). Oxygen concentrations recovered to levels before the [O_2_]-drop events within 10–85 min. In both culms, two to three succeeding [O_2_]-drops were recorded from 19:00 to 21:00 h and between 04:20 and 08:40 h (25 August 2009 culm A1 only). Shortly after an [O_2_]-drop took place in culm A1 on 25 and 26 August 2009, peaks of [O_2_] were detected in rhizome A1. Subsequently to an evening O_2_drop event, [O_2_] in rhizome A1 increased up to 0.1 %, and up to 0.5 % after a morning [O_2_]-drop.

### Plant activity: Gas-exchange of leaf blades

Both *Phragmites* plants A1 and A2 had positive rates of photosynthetic assimilation (A) in the leaf blades when PFD exceeded 100 µmol m^−^^2^s^−^^1^ (Fig. 1). The stomatal conductance (g_s_) was constantly high during the measurement. The rise of A rates above 10 µmol(CO_2_)m^−^^2^s^−^^1^ coincided mainly with the time of culm-[O_2_] increase after sunrise (typically 07:30 h), which shows that the start in plant-internal ventilation begins with the photosynthetic activity of the plant in the morning. The rate of A correlates at least moderately strong during the whole period in shoots (*r* = 0.50, *P* 0.001) and rhizomes (*r* = 0.47, *P* 0.001). The daily correlations showed high variations (see Table 2). The observed correlations between [O_2_] and photosynthesis are in accordance with the moderate correlation to PFD, as photosynthesis is directly linked with the availability of sunlight. During more cloudy and windy conditions on 26 August 2009, rates of photosynthesis above 10 µmol(CO_2_)m^−^^2^s^−^^1^ were reached later (10:00 h) and for shorter periods. Rates of A above 15 µmol(CO_2_)m^−^^2^s^−^^1^ were recorded when PFD exceeded 1600 µmol m^−^^2^s^−^^1^. After 16:00 h, the photosynthetic rate declined and were below 10 µmol(CO_2_)m^−^^2^s^−^^1^ after 18:00 h.

### Carbon dioxide in the culms

**Figure 2.**
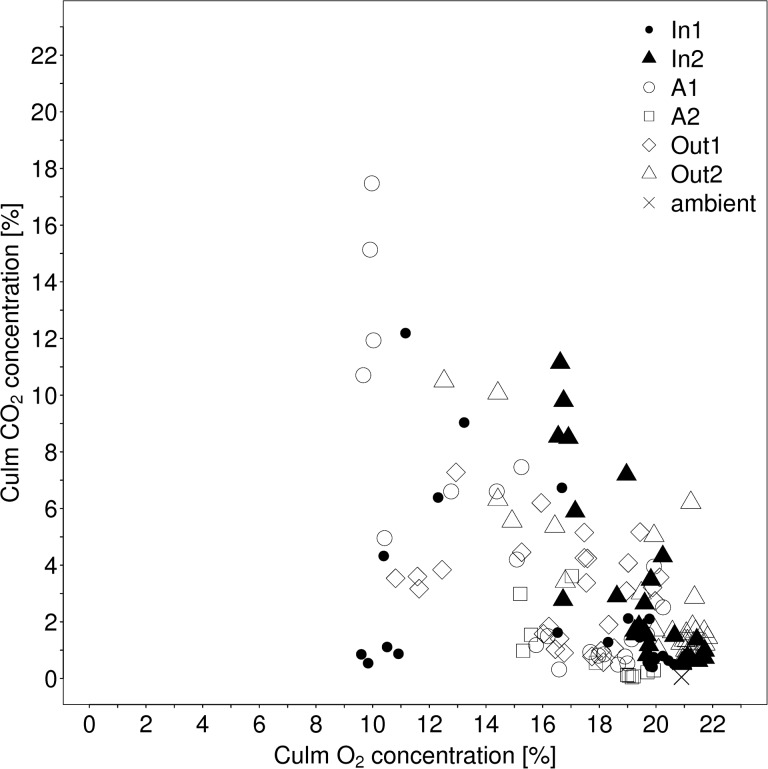
Scatterplot of culm internal oxygen concentration against internal carbon dioxide concentration in the six investigated *P. australis* plants. For correlation coefficients see Table 4.

The ambient levels of [CO_2_] at the study site during stable atmospheric conditions reached maximum levels of 0.050–0.063 % around sunrise (05:00 h), which decreased to 0.038 % during the day (06:00–16:00 h) and subsequently increased over 0.04 %. The [CO_2_] in the culms showed high amplitudes that ranged from 0.32 to 17.5 % (A1; Fig. 1B) and from 0.044 to 3.61 % (A2; Fig. 1C). Maximum internal [CO_2_] were recorded in the early morning hours and ranged in the inflow zone from 11.1 to 12.2 % (plants In1, In2; Fig. 2) and in the outflow zone from 1.55 to 17.5 % (Out1, Out2: 7.28–10.5 %; Fig. 2; A1: 4.96–17.5 %; A2: 1.55–3.61 %; Fig. 1). Starting with the time of [O_2_] increase (after 07:30), internal [CO_2_] decreased to minimum values over midday between 0.40 and 0.52 % in the inflow region (In1, In2) and from 0.04 to 0.94 % in the outflow region (A1, A2, Out1, Out2). After 18:00 h, internal [CO_2_] increased in all culms up to the latest measurement (21:00 h).

On the whole data basis, [CO_2_] were correlated negatively with [O_2_] in the culms (*r* = −0.58, *P* 0.001; Fig. 2). Strong negative correlations were found in five of the six investigated plants: A1 (*r* = −0.84; *P* 0.001), A2 (*r* = −0.7; *P* < 0.05), In1 (*r* = −0.47; *P* < 0.05), In2 (*r* = −0.82; *P* < 0.001) and Out2 (*r* = −0.85; *P* < 0.001), although the courses of [CO_2_] varied according to plant and day. On 25 August 2009, the [CO_2_] in Out1 were constantly high (3 %), while the observed [O_2_] remained below 17.8 %. On the contrary, on 27 August 2009 the [CO_2_] in Out1 were constantly below 2 % and the observed [O_2_] was constantly above 16.7 %, thus indicating some day-to-day variation in plant-internal [CO_2_].

### Modelling nocturnal oxygen courses

**Figure 3.**
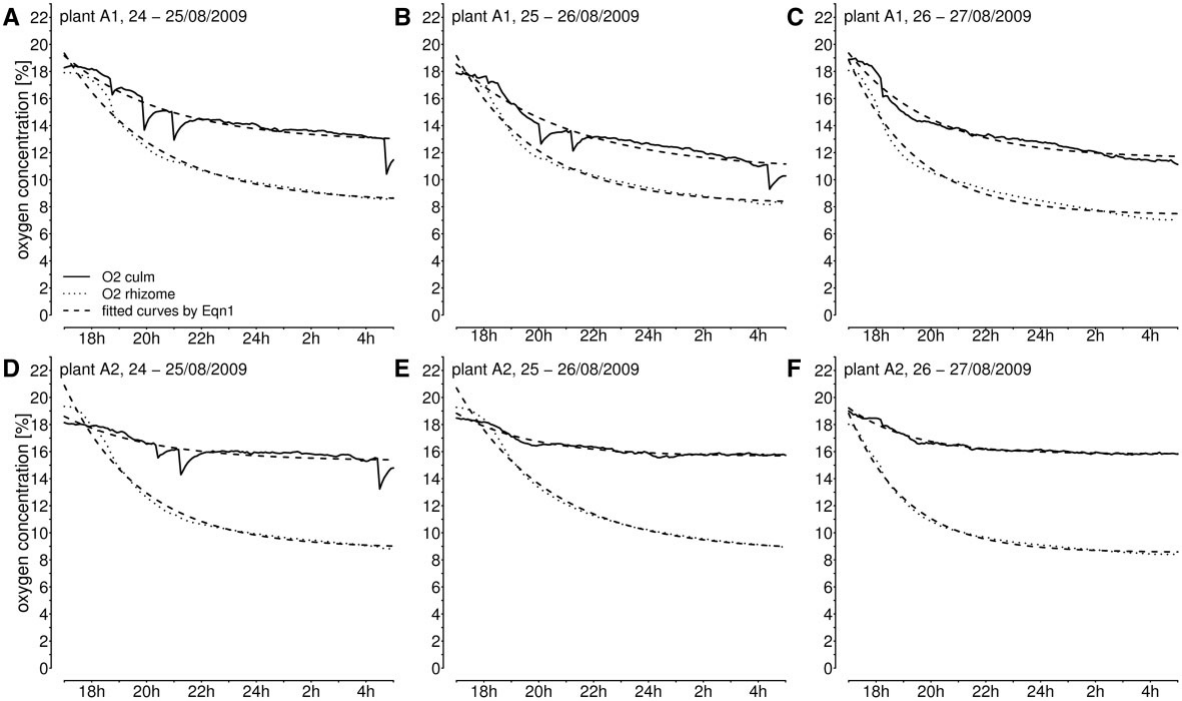
Oxygen concentration recorded overnight from 17:00 h until 5:00 h in culms and rhizomes of *P. australis* plant A1 **(A–C)** and plant A2 **(D–F)**. Exponential curves were fitted with time (t in [s]) against the oxygen courses with the equation (1): $f\left(t\right)=\;a\cdot \exp\left(b\cdot t\right)+\;c$. The coefficients of determination were in all fits above *r*^2^ > 0.9 with *P* < 0.001, except in (E) culm A2 from 25 to 26 August 2009 (*r*^2 ^=^ ^0.76, *P* < 0.001).

The coefficients of determination (*r*^2^) for the exponential Equation (1) modelling the nocturnal courses of [O_2_] in culms and rhizomes were above 0.9 (Fig. 3), except in culm A2 from 25 to 26 August 2009 (*r*^2 ^=^ ^0.76). The fitted intercept (c) was interpreted as the minimum level of internal [O_2_] convergence due to the balance between oxygen demand by aerobic respiration of plant tissues, metabolic activity in the sediment, radial oxygen loss, and oxygen supply through the aerenchyma under the non-through-flow condition. Thus [O_2_] approached a night-time steady-state oxygen level of about 10.7–12.7 % in culm A1, and 15.5–15.7 % in culm A2. In both plants, rhizome-[O_2_] converged to values around 6.7–9.0 %.

The initial declining slopes of [O_2_] varied between −1.1 and −2.8 % h^−^^1^ in the culms and between −3.3 and −4.9 % h^−^^1^ in the rhizomes. Thus, the initial decline was twice as steep as in the rhizomes as in the corresponding culms. Rates of oxygen decrease were apparently reduced in the morning hours (05:00 h) to values between −0.02 and −0.12 % h^−^^1^ in culm A1, and between −0.01 to −0.02 % h^−^^1^ in culm A2. Rhizome-[O_2_] decrease rates varied in the early morning between −0.04 and −0.09 % h^−^^1^ in rhizome A1 and between −0.02 and −0.11 % h^−^^1^ in rhizome A2 and were thus in the same range as in the culms. In summary, the twice as high oxygen consumption in rhizomes compared with the culms in the evening levelled down in rhizomes in early morning to the same rates as in the aerial culms. This indicates that oxygen consumption is down-regulated in the submerged plant organs and the rhizosphere at declining oxygen supply.

### Experimental limitation of oxygen supply

**Figure 4.**
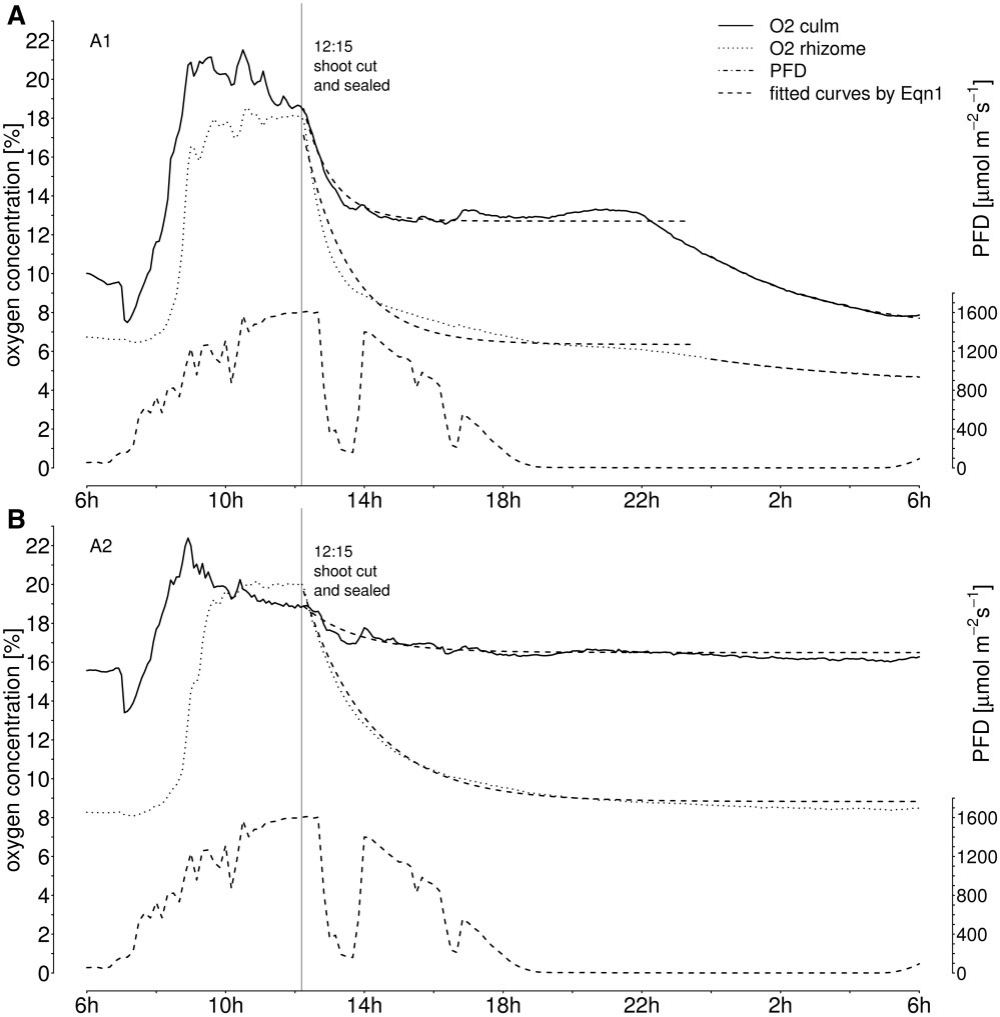
Concentration of internal oxygen in *P. australis* culm stumps after experimental removal of the culms above the implanted oxygen sensor. The pith cavity of the remaining stump was sealed airtight and measurements continued for 20 h overnight. Exponential curves were fitted with time (t in [s]) against the oxygen courses with the equation (1): $f\left(t\right)=\;a\cdot \exp\left(b\cdot t\right)+\;c$; compare to Figure 3. **(A)** plant A1; **(B)** plant A2. The coefficients of determination were in all fits above *r*^2^ > 0.9 with *P* < 0.001.

The analyzed culms A1 and A2 were cut at the internodes above the oxygen sensor insertion on 27 August 2009 at 12:15 h and the stumps were given an airtight seal. The [O_2_] in both culms and rhizomes showed a sudden and steep decrease after cutting (Fig. 4). Clearly, internal [O_2_] decreased despite high PFD and low RH. The initiated effect was similar to, but faster than, the evening decreases observed previously. Initial slopes were −8.4 % h^−^^1^ in culm A1 and −1.5 % h^−^^1^ in culm A2, and from −6.3 to −8.4 % h^−^^1^ in the respective rhizomes. In plant A2, [O_2_] dropped to values in the range of overnight levels 18 h after the culm was cut (culm stump: ∼16.7 %, rhizome: ∼8.4 %). About 12 h after the culm was cut, [O_2_] levels in culm stump and rhizome of plant A1 were also similar to overnight decline. However, during the following 6 h, the internal [O_2_] decreased further to about 7.3 and 5.2 % in culm stump and rhizome, respectively. Thus, the previously observed mechanisms for down-regulation of oxygen consumption were also initiated when oxygen supply was experimentally interrupted but these mechanisms were not sufficient beyond 12 h without new, actively ventilated air.

## Discussion

### Oxygen and carbon dioxide dynamics in culms and rhizomes

Dynamics of oxygen concentration inside wetland plant gas-spaces have been previously studied mainly in laboratory or greenhouse experiments or on excavated plants and culms (Armstrong and Armstrong 1990; Armstrong *et al.* 1992). Studies on intact plant shoot-rhizome systems in the field are rare (Brix *et al.* 1996; Konnerup *et al.* 2011). The diurnal courses of [O_2_] recorded in this study in culms and rhizomes confirm the strong influence of RH on internal oxygen concentration under undisturbed growing conditions, especially in rhizomes (Table 2). This aligns with previous works demonstrating that internal ventilation is mainly driven by humidity-induced convection (Afreen *et al.* 2007; Armstrong and Armstrong 1991; Armstrong *et al.* 1992; Bendix *et al.* 1994; Brix *et al.* 1992, 1996; Dickopp *et al.* 2011; Tornbjerg *et al.* 1994). The effect of air temperature can be explained by increasing air temperature resulting in decreasing RH and higher leaf temperatures which elevated the internal water vapour pressures (Armstrong *et al.* 1992). Thus thermal transpiration supports pressurization efficiency of wetland macrophytes during hours of high PFD (Brix *et al.* 1996; Konnerup *et al.* 2011).

The culm-[O_2_] obviously increased earlier than rhizome-[O_2_], at a similar morning time irrespective of RH or PFD (see Table 3). This shows that the pressurization in the culms started already before low RH was reached and demonstrates the active role of the shoot tissues of *P. australis* for pressurized gas-flow (Afreen *et al.* 2009; Armstrong and Armstrong 2005). The steep slopes of [O_2_] resulted from the widening of stomatal aperture in the early morning, and in the early evening hours from the narrowing of the stomata (cf. Armstrong *et al.* 1992; Brix *et al.* 1996; see also Figure 1 for stomatal conductance). The close correlations between [O_2_] and gas exchange observations confirm that the stable diurnal dynamics of [O_2_] in shoots and rhizomes were highly influenced by the photosynthetic activity of the plant (hypothesis 2). A close relationship between photosynthesis and ventilation was also found for alder (Armstrong and Armstrong 2005). Especially in the morning hours, the start of the gas exchange initiated the internal pressurized gas ventilation.

The difference in levels of [O_2_] between rhizomes and culms during the middle of the day indicates that oxygen-rich air is actively pressurized from culm to rhizome gas-spaces during that period (Brix *et al.* 1996). In the evening rhizome-[O_2_] decreased as soon as RH increased and PFD declined below 100 µmol m^−^^2^s^−^^1^ while culm-[O_2_] remained high for another hour (Fig. 1). Throughout the measurements, there was a significant lag between the oxygen courses in culms and rhizomes. The above-mentioned observations reveal the importance of pressurized gas-flow for rhizome aeration initiated in the culm.

During the night, micro-climatic parameters were unfavourable for pressurization mechanisms and, as a result, ventilation was dominated by diffusion or, if present, by Venturi effects (Afreen *et al.* 2007; Armstrong *et al.* 1992). As pressurization decreased in the evenings, internal [O_2_] declined rapidly. Aerobic respiration of submerged plant organs (Čížková and Bauer 1998), radial oxygen loss (Armstrong *et al.* 2000) and biological and geochemical oxygen demand in the rhizosphere (Dušek *et al.* 2008) deplete oxygen reserves in the submerged organs. Brix *et al.*(1996) proposed that 5–20 % of the oxygen vented through below ground organs of *P. australis* is consumed by oxygen-dependent processes like respiration. The gradual reduction in rhizome-[O_2_] by as much as 50 % of the maximum values indicates very high oxygen demand in the rhizosphere of *Phragmites* plants at this study site.

The [O_2_]-drops observed in the reed culms during the night can be interpreted as sudden, strong effects of convective gas-flow in the culm-rhizome-system. The oxygen optode placed at the end of the rhizome concomitantly registered a slight [O_2_] increase. The [O_2_]-drops might be also related to stomatal widening (Armstrong *et al.* 1992) and narrowing as they occurred in the early morning and evening hours (cf. Fig. 1). Another explanation may be that [O_2_]-drops were the readings of local effects of a Venturi driven through-flow event (Armstrong *et al.* 1992). These sudden changes in internal gas concentrations indicate that there are other gas exchange patterns besides of diffusion and convective gas-flow.

High diurnal variation in [O_2_] was accompanied by changes in [CO_2_] showing strong negative correlations between the concentrations of both gases (cf. Figs. 1 and 2) and thus confirming the hypothesis (1). Obviously, [CO_2_] diffused in to plant tissues and accumulated in the pith cavities during the night when pressurization was inactive. The highest [CO_2_] values were detected at the beginning of the sharp increase in culm-[O_2_], which resembles the flush of CO_2_-rich air from the rhizome system when the through-flow started in the morning (Konnerup *et al.* 2011). Subsequently, [CO_2_] declined steeply, showing that the below ground organs were well supplied with fresh air and the plant-internal CO_2_ was either vented to the atmosphere or utilized in photosynthesis. The negative correlation between plant-internal [O_2_ and CO_2_] in the plant Out1 was significant only in two out of four days, due to high intraday variation. Constantly low levels of [O_2_] resulted in higher [CO_2_] at less diurnal variation ([O_2_] < 17.8 %, [CO_2_] 3 %) on 25 August 2009. Conversely, constantly high [O_2_] was linked to lower [CO_2_] ([O_2_] > 16.7 %, [CO_2_] < 2 %) on 27 August 2009.

High CO_2_ levels in the plant aerenchyma were reported also for *Scirpus lacustris* and *Cyperus papyrus* (Singer et al. 1994) and *T.* *latifolia* (Constable and Longstreth 1994) but were not linked to plant aeration. Such high [CO_2_] can only originate from a combination of all biological processes in the submerged zones including aerobic respiration of rhizomes, roots and rhizosphere microorganisms and anaerobic fermentation processes. Specifically in plant A1, the molar CO_2_/O_2_ ratio exceeded 1, which is typical for respiratory quotients of anaerobic systems (Dilly 2003). In contrast, in the plant In1, situated in the inflow zone of the CW, the [CO_2_] even declined at low culm-[O_2_]. In this part of the CW with high organic load, Dickopp *et al.* (2011) observed low diurnal oxygen dynamics and argued that below ground plant organs might be less permeable in order to prevent diffusion of toxic fermentation products.

Our observations show that it *in* *situ* measurements may reveal conditions, which are difficult to synthesize to an overall explanation as they reflect highly divers settings influencing the individual plants. The below ground rooting system of *P. australis* is very complex and alters in age and size. Herbivory or diseases may cause the disconnection of parts of the rhizomes from the ventilation stream and thus result in differences between the responses of the plants.

### Oxygen supply and consumption

Steep slopes of [O_2_] decline in rhizomes began to decelerate when oxygen concentrations dropped below 14.6 %, and later oxygen levels remained above 6.7 % (cf. Figs. 1 and 3). In buried horizontal rhizomes of the same part of the studied CW the mean minimum rhizome-[O_2_] recorded was around 5.8 ± 1.5 % SD (Dickopp *et al.* 2011). The recorded minimum values in the rhizomes may represent the balance between oxygen supply by diffusion and oxygen demand by submerged plant organs, oxygen loss to the rhizosphere and rhizosphere-related aerobic processes. Also the unmodifiable barriers against radial oxygen loss were presumably more effective at lower oxygen gradients between inner gas lacunae and the surrounding anoxic environment (Armstrong *et al.* 2000). In accordance with our hypothesis (3), the presented observations suggest that plant organs and rhizosphere microorganisms have mechanisms to reduce their rate of oxygen consumption at low oxygen availability, i.e. during night and after culm removal.

Assuming exponential decline is reasonable for the observed [O_2_] courses and reproduces the convergence to minimum oxygen values inside the reed pith cavities at reduced ventilation. The declining oxygen curves (cf. Figs. 1 and 3) agree with the modelled curves of internal oxygen concentration in respiring roots during down-regulation of respiration (Armstrong and Beckett 2011b). Also the soil microorganisms can be assumed to down-regulate their rate of aerobic respiration when the oxygen availability becomes limited (Jin and Bethke 2003), i.e. during periods of reduced plant ventilation. The overall rate of substrate-related oxygen consumption in an undisturbed plant-rhizosphere system is reflecting the plant and microbial oxygen demand, oxygen loss from the roots and biogeochemical oxygen demand in the rhizosphere and may react more sensitively than the above mentioned laboratory studies. Our data indicate that substrate-related oxygen consumption already slowed down when air with around 14 % oxygen was present in the pith cavities.

The excision of the investigated culms at midday, when micro-climatic conditions were generally favourable for pressurization, showed the dependence of oxygen on pressurized gas-flow from upper plant parts (Fig. 4). Removing major parts of the above ground plant organs caused [O_2_] to drop sharply, although the remaining culm stumps had 30 cm of functional leaf sheath area left. The declining oxygen curves after culm-cut show the submerged system is highly dependent on the active ventilation by the culm. In times of active oxygen supply i.e. during midday, the rhizosphere respiration seems to be highest in the presence of ample molecular oxygen, as shown by twice as high initial oxygen consumption after culm cut (−3.3 to −4.6 % h^−^^1^ initial decline in intact plant against −6.3 to −8.4 % h^−^^1^ after the culm cut at midday). The rhizosphere of *P. australis* is intensively inhabited by aerobic methane-oxidizing bacteria (Faußer et al. 2012), which are presumably very active during the period of highest oxygen supply. The minimum levels of [O_2_] indicate that, additionally to plants’ oxygen consumption rate (Armstrong and Beckett 2011b), also rhizobacterial metabolic activity will be triggered by the availability of free oxygen (Jin and Bethke 2003).

The reduction of oxygen levels to about 50 % of predawn values indicates that the rhizome system connected to plant A1 was highly dependent on the experimentally removed shoot. Also the high [CO_2_] of up to 18 % pointed out that there is a large respiratory turnover in the submerged organs and rhizosphere of plant A1. In the plant A2, the [O_2_] levels in culm stump and rhizome approached 18 h after the culm removal similar values to previous morning concentrations. This indicated that plant A2 was connected to an extended rhizome system, which had good ventilation properties, and could compensate better for the loss of the current culm A2 (cf. Čížková and Lukavská 1999).

Hypoxic internal conditions in the submerged organs can be expected when internal [O_2_] converged to levels <6.3 % in culms and 4.2 % in rhizomes (Armstrong *et al.* 2009; Gupta *et al.* 2009). If the root internal oxygen concentrations reach the range of the critical oxygen pressure (i.e. 45 hPa or 3.1 %) respiration will be down regulated (Armstrong *et al.* 2000, 2009; Berry and Norris 1949; Čížková and Bauer 1998; Gupta *et al.* 2009; Zabalza *et al.* 2009). Although the presented pre-dawn oxygen concentrations in the rhizome are above the critical oxygen pressure, down regulation can be expected as oxygen is transported inside the roots by diffusion only. The mechanism of oxygen conservation was modelled for respiring root segments by Armstrong and Beckett 2011b). Down-regulation starts when an anoxic core spreads through the root diameter. Therefore the down-regulation of respiration in apical roots is reflected by slow oxygen decline in rhizome cavities, which serve as oxygen reservoir for root and rhizosphere respiration. These results support the hypothesis (3) that demand for oxygen is regulated during periods of low oxygen availability in submerged regions.

## Conclusions

Common reed (*P. australis*) has a high capacity to actively ventilate oxygen-rich air to submerged organs and to the rhizosphere. The relationship between internal oxygen and carbon dioxide concentrations measured under field conditions reflects the dependence of respiration of the submerged organs and biogeochemical processes in the rhizosphere on plant-internal oxygen supply. Regulation of oxygen demand within an intact rhizosphere was observed for the first time in the field and is highly relevant for the understanding of processes in submerged parts of wetland ecosystems.

The obviously reverse dynamics of oxygen and carbon dioxide concentrations reveal a close relationship between the supply of oxygen by the plant and the rhizosphere-related production of CO_2_. The slopes of declining oxygen concentrations in culms and rhizomes during the night and in particular after culm-cut notably demonstrated the regulation of the oxygen consumption for the complete below ground plant-associated system. This underlines the importance of field studies for a better understanding of sediment aeration processes. The oxygen released by roots of wetland plants is readily utilized for oxidative processes (Becket et al. 2001, Bodelier 2003, Faußer et al. 2012) and the metabolic activity of the rhizospheric microbiota is regulated in dependence on available oxygen. Field research on the oxygen-carbon dioxide interrelationship will provide new and globally important data for carbon balance and methane emissions from wetlands.
